# Impact of cold air exposure on respiratory physiology during light‐to‐moderate physical activity in healthy adults

**DOI:** 10.14814/phy2.70801

**Published:** 2026-03-03

**Authors:** Christopher L. Chapman, Adam W. Potter, Erica A. Schafer, Karl E. Friedl, J. Luke Pryor, David P. Looney

**Affiliations:** ^1^ Thermal and Mountain Medicine Division US Army Research Institute of Environmental Medicine Natick Massachusetts USA; ^2^ Oak Ridge Institute for Science and Education Oak Ridge Tennessee USA; ^3^ Center for Research and Education in Special Environments, Department of Exercise and Nutrition Sciences University at Buffalo Buffalo New York USA; ^4^ Maximize Human Performance, LLC Framingham Massachusetts USA; ^5^ CoachMePlus Buffalo New York USA

**Keywords:** cold exposure, exercise, heart rate, occupational work, respiration, RPE, tidal volume, ventilation

## Abstract

Working in cold environments presents unique physiological challenges, and this study sought to investigate the specific impacts of cold air exposure on ventilation during light‐to‐moderate physical activity simulating occupational work demands. Fourteen healthy adults (3 females; age: 24 ± 6 years) completed five 20‐min treadmill walking bouts across three environmental conditions (20°C, 10°C, and 0°C) in a randomized, crossover design. Respiratory variables, including minute ventilation (VE), breathing frequency (RR), and tidal volume (Vt), were measured, as well as heart rate (HR) and ratings of perceived exertion (RPE). Data were analyzed using repeated‐measures ANOVA. VE was significantly higher at 0°C compared to both 10°C (mean difference [Δ]: +2.08 L·min^−1^, *p* < 0.001) and 20°C (Δ: +1.62 L·min^−1^, *p* < 0.001) during the final exercise stage. This increased ventilation was primarily driven by a significant increase in Vt at 0°C compared to 10°C (Δ: +0.11 L, *p* = 0.002) and 20°C (Δ: +0.13 L, *p* = 0.002). RR and RPE did not differ between conditions, while HR was modestly affected by temperature. Cold air exposure significantly alters ventilatory patterns during light‐to‐moderate physical activity. Increased ventilation observed at colder temperatures is primarily mediated by increases in Vt. These findings provide insight into acute cold‐air respiration and highlight implications for performance in austere environments.

## 1 INTRODUCTION

The unprotected respiratory system is particularly susceptible to acute cold air exposure, posing significant physiological challenges, especially during physical activity. Inhaling cold air can trigger a cascade of events, including increased airway resistance, bronchoconstriction, and alterations in the osmolarity of the airway lining fluid (Giesbrecht, 1995). This increased osmolarity can lead to histamine release, potentially contributing to increased vascular permeability and epithelial damage in the lungs (Findlay et al., 1981). Moreover, cold air inhalation can exacerbate exercise‐induced asthma (EIA) or induce bronchospasm in susceptible individuals (Rundell et al., 2015; Rundell & Jenkinson, 2002). The severity of EIA is directly related to the temperature and humidity of the inhaled air, with colder, drier air increasing the risk and severity of bronchoconstriction (Rundell & Jenkinson, 2002). Reduced baseline minute ventilation and respiratory chemosensitivity have also been observed with cold air exposure (Giesbrecht, 1995; Kovtun & Voevoda, 2013). Another risk from continuous cold air breathing is a progressive cooling of the whole body via the lungs and potential hypothermia (Giesbrecht, 1995). Beyond the direct effects on the airways, the metabolic cost of breathing cold air can be substantial, as the body expends energy to warm and humidify the inspired air (Giesbrecht, 1995; Van Ooijen et al., 2004). This increased energy demand and potential for airway irritation may lead to compensatory respiratory adjustments to maintain efficient gas exchange, such as increasing tidal volume to reduce dead space ventilation. This energy expenditure can further strain physiological resources during prolonged physical activity, potentially impacting performance.

Cold air exposure causes an integrative physiological stress to humans, including the respiratory system, that can impair physical performance during military operations and outdoor recreational activities (e.g., hiking, mountaineering) (Giesbrecht, 1995; Schafer et al., 2024). During inspiration, the cold environmental air is warmed and humidified, resulting in dehydration of the respiratory epithelium that is exacerbated by colder temperatures (Strauss et al., 1977) and water vapor in the air (Deal Jr et al., 1979), when switching from nasal to mouth breathing (Chand et al., 1986; Deal Jr et al., 1979; Griffin et al., 1982), and/or increasing ventilation (McFadden et al., 1982), especially during exercise (Giesbrecht, 1995). Cooling of the airways, body core, and skin reduces respiratory chemosensitivity and baseline ventilation (Guyenet, 2014; O'regan & Majcherczyk, 1982). Prolonged exposure to cold air may elicit bronchoconstriction, shortness of breath, and hyperventilation thereby increasing the metabolic demands of the respiratory muscles for more forcefully and rapid contractions (Hinde et al., 2017). These responses may negatively impair physical performance and increase risks of adverse health effects to jeopardize mission success for military personnel and athletes alike (Gleeson & Pyne, 2016). Effective countermeasures for mitigating undesired effects of cold air exposure, such as reducing exercise intensity or mouth covering (Kennedy & Faulhaber, 2018), are not always feasible, especially for military personnel.

Military personnel and recreational athletes may encounter cold air temperatures in austere and dynamic environments where route selection is complicated by bulky cold weather clothing (Potter et al., 2018, 2023) and inclement winter weather that further heighten the energetic costs of traversing (Looney et al., 2025; Richmond et al., 2019). Recent work has shown that steeper but shorter routes can minimize metabolic cost in cold environments (i.e., “steeper is cheaper”) (Looney et al., 2025); however, the respiratory demands are greater, and implications of this strategy remain unclear.

This study aimed to quantify the physiological impact of progressive cold strain on respiratory responses during light‐to‐moderate intensity treadmill exercise. We hypothesized that cold air exposure to progressively colder ambient temperatures (20°C, 10°C, and 0°C) would alter respiratory patterns during exercise, and we aimed to quantify these changes in minute ventilation, tidal volume, and breathing frequency (respiratory rate). We investigated these hypotheses by measuring ventilation, breathing frequency, tidal volume, and pulse‐respiration quotient under controlled laboratory conditions.

## 2 METHODS

### 2.1 Ethical approval

The Institutional Review Board at the US Army Medical Research and Development Command (MRDC; Fort Detrick, MD, USA) approved this study. Researchers adhered to the principles of the Declaration of Helsinki, Department of Defense Instruction 3216.02, and 32 CFR 219 on the use of volunteers in research. All participants were briefed on the study and its potential risks prior to providing their written informed consent.

### 2.2 Participants

Fourteen healthy adults (3 females and 11 males; age: 24 ± 6 years; height: 172 ± 8 cm; body mass: 72.0 ± 16 kg, body fat: 21.5 ± 8.2%, body surface area: 1.8 ± 0.2 m^2^) participated in this study, including 10 active‐duty US Army Soldiers (3 females) and four male civilians. The sample size was chosen based on feasibility and is comparable to similar physiological studies in this field. Participants were screened by a physician and were eligible for the study with ages between 18 and 44 years and having a minimum physical activity of 2 days per week of aerobic or resistance exercise for at least 30 min. Exclusion criteria for the study included: (1) musculoskeletal injuries; (2) pregnancy; (3) claustrophobia or difficulty breathing into a mouthpiece; (4) history of frostbite, exercise‐induced asthma (EIA), cold‐induced asthma, Raynaud's syndrome, and/or non‐freezing cold injuries; (5) contraindications to the telemetry pill used to measure core temperature as described by the manufacturer; (6) current use of serotonergic, diuretic, myopathic, or nephrotoxic medication, furosemide, and anticoagulant medication.

### 2.3 Experimental design and procedures

The present study employed a randomized, crossover design where individuals participated in one familiarization visit, one baseline visit, and three experimental visits where the environmental conditions were randomized (20°C, 10°C, and 0°C) (hereafter referred to as temperate (20°C), cool (10°C), and cold (0°C) conditions, respectively). The physical performance battery and resting metabolic rate data were collected for the parent study and are reported elsewhere (Chapman et al., 2024; Looney et al., 2024; Looney et al., 2025); the present analysis focuses exclusively on the cardiorespiratory responses during treadmill walking. All visits were separated by ≥48 h to ensure sufficient recovery and mitigate any potential influence from previous visits. Visits for all participants commenced between 0500 and 0700 h and at the same morning time for a given participant to control the time of day of testing. Participants refrained from (1) high‐intensity exercise for >48 h; (2) alcohol for >24 h; and (3) caffeine, nicotine, and food intake for ≥10 h. Euhydration upon arrival to a study visit was encouraged by instructing participants to consume ≥500 mL water both the night before and the morning of each visit. Participants provided a urine sample upon arrival to all visits to ensure urine specific gravity was ≤1.030 to increase the generalizability of our findings given the high prevalence of mild dehydration in adults (Chapman et al., 2023).

The larger protocol aimed to quantify the physiological and performance responses to progressive cold strain during prolonged light‐to‐moderate intensity physical work in cold environments using laboratory‐controlled conditions. To ensure sufficient cold strain was induced during this experimental paradigm, participants wore combat boots and standard light physical training attire (i.e., shorts, t‐shirt, and socks or the Army Physical Fitness Uniform), because the minimal operating air temperature of the climatic chamber was 0°C. Participants also wore lightweight (88% polyester and 12% spandex), commercially‐available, running gloves (B07WNM4FXW, CEVAPRO, Amazon, Seattle, WA) on their hands to improve comfort over the duration of the study. Modeling performed during the study design phase indicated that the selected clothing at the environment temperatures of 20°C, 10°C, and 0°C would provide an optimal response scenario (Potter et al., 2020; Xu et al., 2021).

The familiarization (visit 1) and baseline (visit 2) visit procedures were the same. Resting metabolic rates were measured for 30 min as previously described (Chapman et al., 2024; Looney et al., 2025). Then, participants entered the climate‐controlled chamber (20.3 ± 0.7°C and 49 ± 5% relative humidity) and performed one round of a battery of physical performance tasks, followed by 20 min of graded treadmill walking, and then one final round of the performance battery. These activities simulated occupational work demands as testing an array of maximal strength and dexterity performance indicators and light‐to‐moderate physical activity. The performance battery included the following workflow: (1) three static squat jumps with 30 s rest between each repetition; (2) three maximal effort isometric mid‐thigh pulls for ~3–4 s with 30 s rest between each repetition; (3) Complete Minnesota Manual Dexterity Test (Model 32023A, Lafayette Instrument, Lafayette, IN); (4) three repetitions on each hand of standing maximal effort isometric handgrip strength testing with 15 s between each repetition; and (5) perceptual measures (e.g., thermal sensation). The performance battery was immediately followed by 20 min of treadmill walking that included five 4‐min stages at ascending stages of treadmill walking. The intensity of these stages was defined by vertical speed (treadmill speed grade), a measure of vertical ascent rate, with values of 0.00, 1.93, 3.86, 5.79, and 7.72 m·min^−1^ that were randomized as described elsewhere (Looney et al., 2025). Upon finishing the treadmill walk, participants completed the performance battery one more time following the same previously described procedures.

The three experimental visits (visits 3–5) differed only by the environmental temperature (20°C, 10°C, and 0°C), which were randomized between participants. After providing written confirmation of adherence to the protocol restrictions, participants were instrumented. Then, participants entered the climatic chamber. Participants were allowed to drink water ad libitum throughout the experiment. The measured conditions of the climatic chamber for each trial were 20°C: 20.3 ± 0.3°C and 50 ± 4% relative humidity, 10°C: 9.8 ± 0.2°C and 56 ± 5% relative humidity, 0°C: 1.3 ± 1.4°C and 40 ± 7% relative humidity with air circulating at ~1.3 m·s^−1^. Upon entering the chamber, participants completed four rounds of performing one performance battery (~10 min) and one 20 min graded treadmill walk using the same sequence described for the familiarization and baseline testing visits.

### 2.4 Measurements and instrumentation

Height was measured using a stadiometer (Model 213, Seca, Hamburg, Germany) on visit 1. Nude body mass was measured with a standard calibrated scale (Model 876, Seca, Hamburg, Germany) on every visit. Body surface area was measured by three‐dimensional body scans (SS20 Booth Scanner, Size Stream LLC; Cary, NC) and body composition was assessed by dual‐energy X‐ray absorptiometry (DPX‐IQ, Lunar Corporation, Madison, WI) on visit 2. Core temperature was measured using a telemetry pill (eCelsius Performance capsule, BodyCAP, Herouville‐Saint‐Clair, France) as a rectal suppository (*n* = 13) or swallowed the night before the study visit (*n* = 1). Both rectal and ingested telemetry pills have been shown to be valid methods for assessing core body temperature during exercise and thermal stress (Casa et al., 2007). Skin temperature was measured via wireless temperature sensors (Thermochron iButton model DS1922L, Maxim Integrated, San Jose, CA) with a resolution of 0.0625°C sampling every 60 s affixed to the skin at the chest, upper arm, thigh, and calf to calculate mean skin temperature using the 4‐site weighting as described by Ramanathan (Ramanathan, 1964). Heart rate was measured using a Polar chest strap (H10, Polar Electro, Kempele, Finland). Expiratory gases were analyzed for minute ventilation, breathing frequency, and tidal volume by fitting a two‐way oro‐nasal breathing mask (7450 series V2; Hans Rudolph Inc.; Shawnee, KS) to a laboratory metabolic cart (TrueMax 2400; ParvoMedics, Sandy, UT). Per manufacturer instructions, the heated pneumotachometer and metabolic cart were warmed up for over 60 min before testing with a minimum of two flowmeter and gas analyzer calibrations. The metabolic cart itself remained outside the environmental chamber in temperate conditions. Per the manufacturer, the pneumotach is precise for detecting changes in volume of this magnitude. The system was calibrated before each test using a 3‐L syringe and gases of known concentration. Ratings of perceived exertion (RPE) was measured using a standard 6–20 Borg scale.

### 2.5 Data and statistical analysis

Data are reported as the steady state 1‐min average taken over the last minute of each walking stage. A metabolic steady state was identified as a respiratory exchange ratio (RER) within the normal respiratory quotient range of 0.7–1.0 and a coefficient of variation of <10% (Holdy, 2004) between the first and second 30 s of the final minute oxygen uptake (*V̇*O_2_) and carbon dioxide production (*V̇*CO_2_) (Looney et al., 2022).

Data were analyzed using R (Version 4.4.1; R foundation for Statistical Computing; Vienna, Austria). Physiological outcomes (minute ventilation (VE), heart rate (HR), breathing frequency (respiratory rate) (RR), and tidal volume (Vt)) from the final‐stage of each temperature exposure were analyzed using a repeated‐measures model. To test our directional hypotheses, we use a priori planned contrasts (0°C vs. 10°C, 0°C vs. 20°C, and 10°C vs. 20°C), an approach that does not require a significant omnibus *F*‐test. For our primary analysis, we report paired mean differences (Δ) with 95% confidence intervals (CIs) and *p*‐values for each contrast. For descriptive purposes, the omnibus *F*‐test and partial eta‐squared (ηp^2^) from the overall model are also presented. Data are reported as mean ± SD, and statistical significance was accepted at *p* < 0.05.

Relationships between vertical speed (speed × grade) and ventilatory parameters were evaluated using linear regression, with slopes compared across temperature conditions to test whether cold exposure altered the rate of ventilatory increase.

Planned comparisons were conducted to address the primary study aims. Outliers were screened for by visually inspecting the experimental data and using the most conservative (± 3) median absolute deviation from the median approach (Leys et al., 2013).

## 3 RESULTS

**FIGURE 1:**
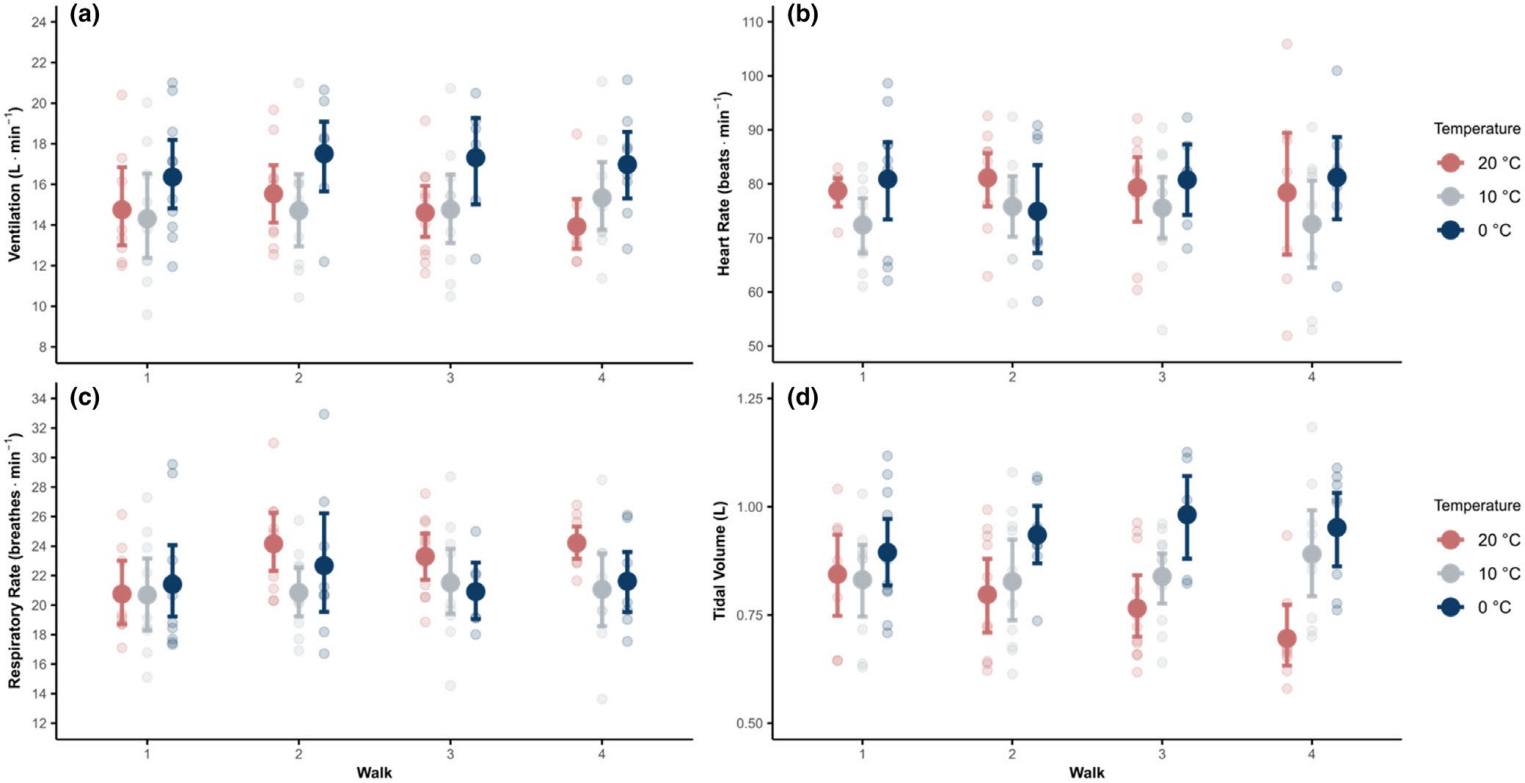
Isolation of cold effects shown in comparison of minute ventilation (VE) (a, top left), heart rate (HR) (b, top right), breathing frequency (respiratory rate) (RR) (c, bottom left), and tidal volume (Vt) (d, bottom right) for all of the level walking trials (32.4 m·min^−1^ at 0% incline) by walk number (1–4), at air temperatures of 20°C, 10°C, and 0°C. Data are presented as mean ± 95% CI.

**FIGURE 2:**
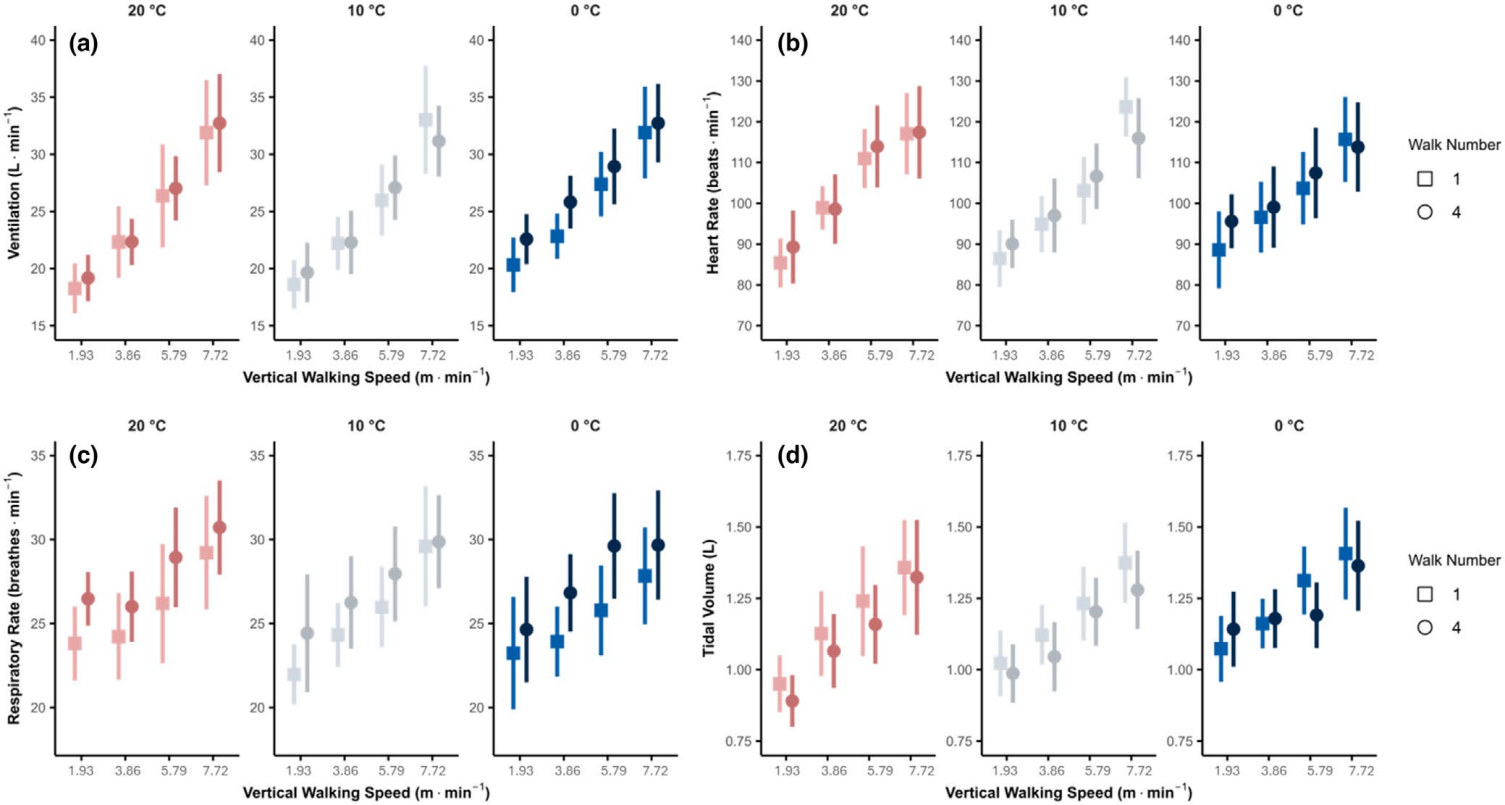
Comparison of increased velocity walking trials (stages 2–5) between walks 1 and 4 for min ventilation (VE) (a, top left), heart rate (HR) (b, top right), breathing frequency (respiratory rate) (RR) (c, bottom left), and tidal volume (Vt) (d, bottom right), at air temperatures of 20°C, 10°C, and 0°C. Data are presented as mean ± 95% CI.

Figure 1 outlines the impact of ambient temperature on key respiratory and cardiovascular variables (VE (Figure 1a), HR (Figure 1b), RR (Figure 1c), and Vt (Figure 1d)) during level walking (32.4 m·min^−1^, 0% incline). This figure provides a clear visualization of the isolated effects of cold air exposure on these physiological parameters during controlled, low‐intensity exercise. Figure 2 compares the responses of VE (Figure 2a), HR (Figure 2b), RR (Figure 2c), and Vt (Figure 2d) during increased velocity walking trials (stages 2–5) between walk 1 and walk 4, across the three ambient temperatures (20°C, 10°C, and 0°C), highlighting influence of increased exercise intensity and varying air temperatures affects these physiological variables.

**FIGURE 3:**
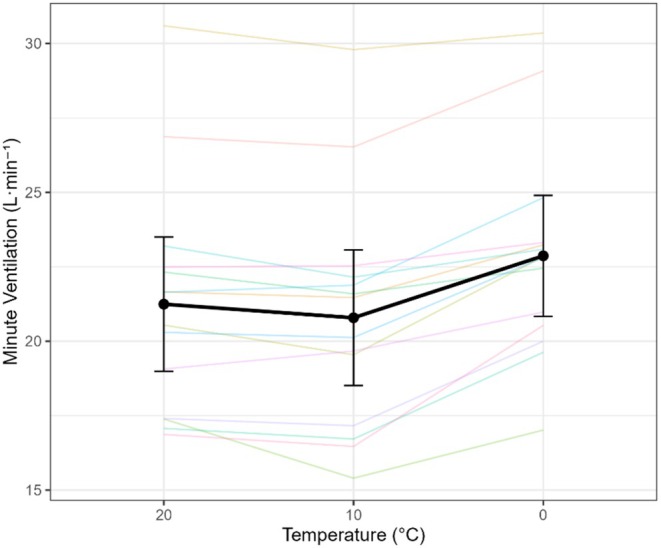
Minute ventilation (VE) in response to varying air temperatures. Spaghetti plot showing individual subject responses and mean VE ± 95% confidence interval across temperatures of 20°C, 10°C, and 0°C. Data shown are from the final minute of the highest exercise stage for each condition.

Minute ventilation (VE) increased significantly as ambient temperature decreased (omnibus *F*(2,68) = 4.17, *p* = 0.02, η^2^ *p* = 0.69). Planned contrasts (Table 1) confirmed that VE was higher at colder temperatures: 0°C vs. 10°C, Δ = +2.08 L·min^−1^ (95% CI: 1.45–2.71, *p* < 0.001); 0°C vs. 20°C, Δ = +1.62 L·min^−1^ (0.85–2.40, *p* < 0.001); and 10°C vs. 20°C, Δ = −0.46 L·min^−1^ (−0.82 to −0.09, *p* = 0.019). These findings demonstrate that ventilation was highest in the cold condition (0°C). Interestingly, ventilation in the temperate condition (20°C) was slightly higher than in the cool condition (10°C) (Figure 3). Linear regression analysis of minute ventilation against vertical speed revealed steep slopes in all conditions, but there was no significant difference between the slopes across the three temperatures (*p* > 0.05), suggesting that the rate of ventilatory increase per unit of work did not change with temperature.

**TABLE 1:** Summary of contrasts.

Outcome	Contrast	Mean ± SD	95% CI	*p*
VE	0 and 10°C	2.08 ± 1.10	1.44 to 2.71	<0.001
VE	0 and 20°C	1.62 ± 1.34	0.85 to 2.40	<0.001
VE	20 and 10°C	−0.46 ± 0.64	−0.82 to −0.09	0.019
RR	0 and 10°C	0.28 ± 2.20	−0.99 to 1.55	0.640
RR	0 and 20°C	−0.53 ± 3.06	−2.29 to 1.24	0.531
RR	20 and 10°C	−0.81 ± 2.74	−2.39 to 0.77	0.290
Vt	0 and 10°C	0.11 ± 0.10	0.05 to 0.17	0.002
Vt	0 and 20°C	0.13 ± 0.13	0.05 to 0.20	0.002
Vt	20 and 10°C	0.02 ± 0.11	−0.04 to 0.08	0.549
HR	0 and 10°C	2.74 ± 5.56	−0.47 to 5.95	0.088
HR	0 and 20°C	−1.40 ± 5.81	−4.76 to 1.95	0.383
HR	20 and 10°C	−4.14 ± 6.53	−7.91 to −0.37	0.034

**FIGURE 4:**
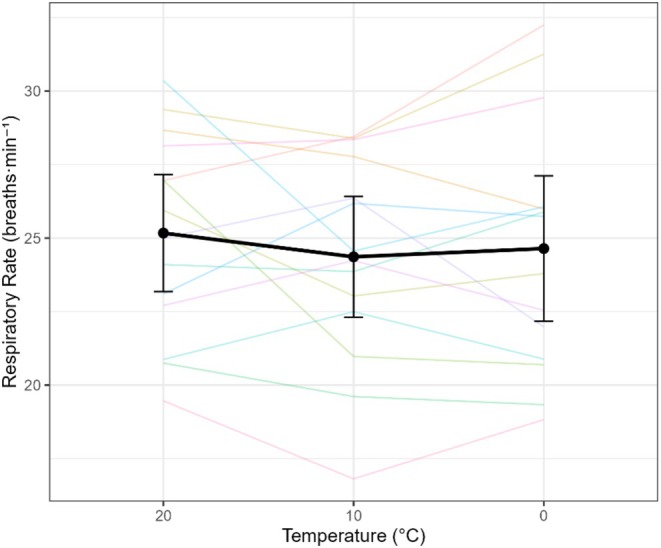
Breathing frequency (respiratory rate) (RR) in response to varying air temperatures. Spaghetti plot showing individual subject responses and mean RR ± 95% confidence interval across temperatures of 20°C, 10°C, and 0°C. Data shown are from the final minute of the highest exercise stage for each condition.

Breathing frequency (RR) was unaffected by temperature (omnibus *F*(2,68) = 0.67, *p* = 0.51, η^2^p = 0.05). Pairwise differences were not significant: 0°C vs. 10°C, Δ = +0.28 breaths·min^−1^ (−0.99 to 1.55, *p* = 0.64); 0°C vs. 20°C, Δ = −0.53 (−2.29 to 1.24, *p* = 0.53); 10°C vs. 20°C, Δ = −0.81 (−2.39 to 0.77, *p* = 0.29). This stability in RR suggests that the cold‐related increase in ventilation was driven primarily by changes in tidal volume (Figure 4).

**FIGURE 5:**
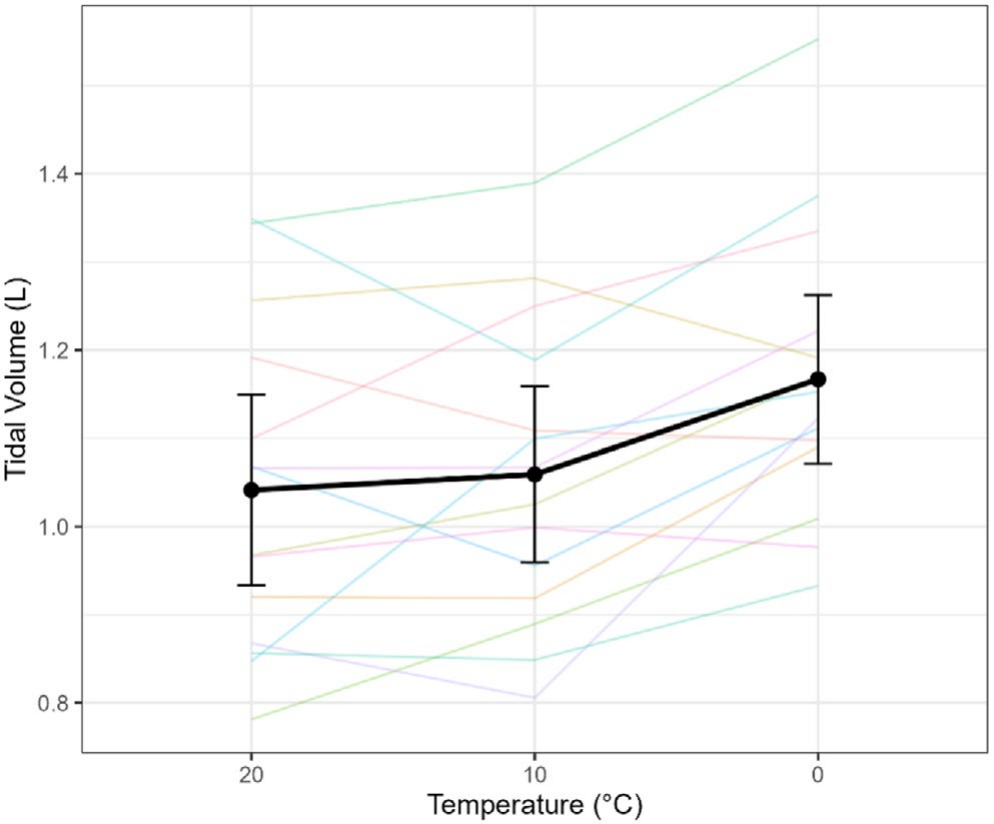
Tidal volume (Vt) in response to varying air temperatures. Spaghetti plot showing individual subject responses and mean Vt ± 95% confidence interval across temperatures of 20°C, 10°C, and 0°C. Data shown are from the final minute of the highest exercise stage for each condition.

Tidal volume (Vt) showed a robust main effect of temperature (omnibus *F*(2,68) = 3.31, *p* = 0.04, η^2^p = 0.44). Planned contrasts indicated greater Vt in the cold: 0°C vs. 10°C, Δ = +0.11 L (0.05–0.17, *p* = 0.002); 0°C vs. 20°C, Δ = +0.13 L (0.05–0.20, *p* = 0.002); 10°C vs. 20°C, Δ = +0.02 L (−0.04 to 0.08, *p* = 0.55) (Table 1). Thus, increases in VE at colder temperatures were primarily mediated by deeper breaths (Figure 5).

**FIGURE 6:**
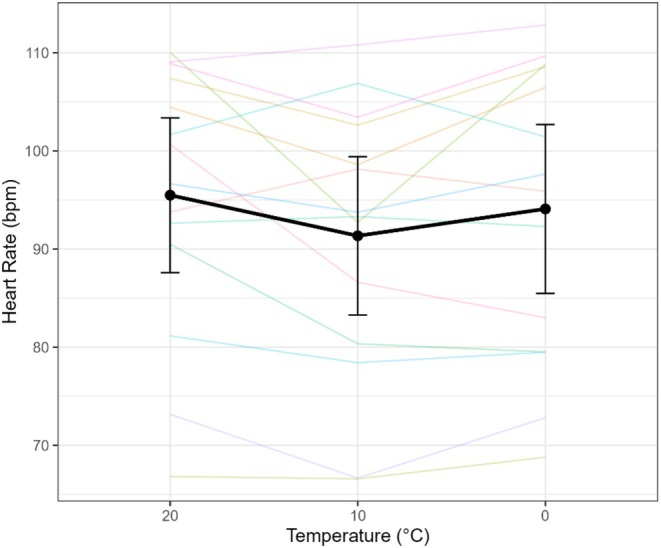
Heart rate (HR) in response to varying air temperatures. Spaghetti plot showing individual subject responses and mean HR ± 95% confidence interval across temperatures of 20°C, 10°C, and 0°C. Data shown are from the final minute of the highest exercise stage for each condition.

Heart rate (HR) differed by temperature (omnibus *F*(2,68) = 3.46, *p* = 0.037, *η* ^2^p = 0.21). Planned contrasts showed a modest, non‐significant increase at 0°C compared to 10°C (Δ = +2.74 bpm, −0.47 to 5.95, *p* = 0.088) and no difference between 0°C and 20°C (Δ = −1.40 bpm, −4.76 to 1.95, *p* = 0.38). However, HR was lower at 10°C than 20°C (Δ = −4.14 bpm, −7.91 to −0.37, *p* = 0.034) (Figure 6). RPE was not significantly different across temperatures (*F*(2,68) = 0.48, *p* = 0.619) but increased significantly with increasing walking speed (*F*(1,68) = 7.51, *p* = 0.008), from 7 ± 1 at stage 1 to 11 ± 2 at stage 5.

Core temperature remained relatively stable across all conditions (*p* > 0.05) (0°C: 37.1 ± 0.3°C, 10°C: 37.2 ± 0.2°C, 20°C: 37.3 ± 0.2°C). Mean skin temperature decreased significantly with decreasing air temperature, with the lowest values observed during the 0°C condition (0°C: 28.1 ± 1.8°C, 10°C: 30.2 ± 1.2°C, 20°C: 32.4 ± 0.9°C; *p* < 0.05). Finger temperature also decreased significantly with decreasing air temperature (F (2,26) = 31.40, *p* < 0.001; 0°C: 26.3 ± 3.1°C, 10°C: 28.4 ± 2.5°C, 20°C: 30.7 ± 1.9°C), further confirming cold strain.

## 4 DISCUSSION

We found that cold air exposure altered ventilatory patterns during light‐to‐moderate physical activity, specifically increasing ventilation as a result of increased tidal volume. It is important to note that the exercise intensities in this study were light‐to‐moderate, and respiratory exchange ratio (RER) values remained below 1.0, indicating that participants were exercising below their ventilatory threshold. Our findings are therefore specific to sub‐threshold exercise and may not apply to high‐intensity work where additional factors drive ventilation. Early work by Keatinge and Evans (Keatinge & Evans, 1961) showed proportional increases in VE and VO_2_ with cold exposure during exercise. Our study builds on this by dissecting the ventilatory response, demonstrating that the increase in VE during light‐to‐moderate exercise in the cold is not achieved by increasing breathing frequency, but rather by increasing tidal volume. This specific strategic shift, deeper, not faster, breaths, is the primary novel finding of our work and highlights an efficient mechanism for preserving alveolar ventilation. These findings align with previous research demonstrating that cold air inhalation stimulates pulmonary receptors, upper airway receptors, and alters peripheral chemoreceptor function, ultimately influencing the central nervous system's control of respiration (Giesbrecht, 1995). Cold‐dwelling mammals such as reindeer and muskoxen have unique adaptations with elaborate nasal scrolled structures that warm and humidity inspired air and reclaim much of the water and heat in a countercurrent flow during exhalation (Blix, 2016). However, these adaptations for warming inspired air may not be advantageous in hot environments, and human physiological regulation of breathing may permit exploitation of a wider range of environmental extremes, including the change in tidal volume in the cold that we observed.

In the cold (0°C) trial, from the third stage (5.79 m·min^−1^) to the fourth stage (7.72 m·min^−1^), increases in ventilation are due to increased breathing frequency (RR) as tidal volume appears to plateau. In stage five (7.72 m·min^−1^), ventilation increases further due to increased tidal volume as breathing frequency plateaus (Figure 2).

The observed increase in ventilation driven primarily by tidal volume at colder temperatures aligns well with established physiological principles. Specifically, inhalation of cold air appears to engage pulmonary receptors and peripheral chemoreceptors, like cold‐sensitive Transient Receptor Potential (TRP) channels such as TRPM8 (active across the range of temperatures tested) and TRPA1 (active at the lowest temperature), prompting the body to increase tidal volume as a compensatory mechanism. This strategy likely minimizes airway resistance and helps maintain effective alveolar ventilation even in challenging cold environments. These responses suggest that in cold environments, breathing strategy shifts toward optimizing breath depth rather than frequency, potentially reducing the risk of hyperventilation and maintaining alveolar gas exchange. This insight enriches our understanding of the broader physiological principles governing respiratory adaptation and has direct implications for individuals working or performing in cold environments.

The observed increase in ventilation at colder temperatures can be attributed to several factors. First, cold air can cause bronchoconstriction, increasing airway resistance and stimulating irritant receptors in the airways (Anderson & Kippelen, 2010). This stimulation can lead to an increase in ventilation in an attempt to maintain adequate oxygen delivery. Second, the increased osmolarity of the airway lining fluid in cold air, as mentioned earlier, can contribute to inflammation and further stimulate ventilation (Deal Jr et al., 1979). The increased tidal volume, specifically, may be a compensatory mechanism to reduce dead space ventilation and improve alveolar ventilation in the face of increased airway resistance. Why does breathing frequency plateau at colder temperatures? Possible explanations include: increased work of breathing associated with higher breathing rates in cold air; central nervous system regulation prioritizing tidal volume to improve gas exchange efficiency; the need to prevent hyperventilation‐induced hypocapnia.

The observed differences in ventilatory responses to increasing walking speed across different temperatures suggest an acute respiratory strategy based on environmental conditions and exercise intensity. Specifically, at 0°C, the initial rise in ventilation was associated with increases in both breathing frequency and tidal volume. However, at higher speeds, breathing frequency plateaued, and further increases in ventilation were primarily driven by tidal volume. This plateau in breathing frequency might indicate a physiological constraint on increasing breathing frequency in cold air, potentially due to the increased work of breathing or a risk of hyperventilation‐induced hypocapnia.

This study has some limitations, including the relatively small sample size, which may limit the generalizability of our findings to others and the statistical power to detect smaller effects. Another limitation is that we did not statistically control for metabolic rate (e.g., via ANCOVA), which may have contributed to the observed changes in ventilation. Although exercise intensity was matched, colder temperatures can increase metabolic rate. Therefore, we cannot completely isolate the effect of temperature on ventilatory control from the effect of temperature on metabolic demand. Future studies should use an ANCOVA approach to partition these effects. This interpretation is supported by observations within our own data; for instance, at some exercise intensities, higher heart rates, a surrogate for metabolic load, corresponded with higher ventilation, reinforcing the need to disentangle the direct thermal effects from the metabolic effects in future work. Furthermore, we calculated mean skin temperature using the 4‐site Ramanathan weighting, which is appropriate for assessing whole‐body heat exchange but may not accurately represent the afferent sensory input from cold‐sensitive neurons, which are not distributed in proportion to surface area. Finally, our protocol required participants to use a mouthpiece and noseclip, enforcing oral breathing. This prevents the natural warming and humidification of air that occurs during nasal breathing, a common real‐world behavior, and thus may have magnified the respiratory responses to cold air. The use of a homogeneous sample of young, healthy adults may also limit generalizability to other populations, and future research should investigate the effects of rewarming on respiratory function.

## 5 CONCLUSION

This study reveals a key acute respiratory adjustment to cold, where the body prioritizes deeper breaths (increased tidal volume) over a faster breathing frequency to increase ventilation. While the observed mean difference in tidal volume of ~100–130 mL may seem small, it represents a ~10% increase from the warmer conditions. This strategic shift to deeper breaths could be practically meaningful during prolonged occupational tasks in the cold by improving ventilatory efficiency and reducing the relative contribution of dead space ventilation. These insights have important implications for maintaining respiratory efficiency in occupational and military settings in cold climates.

## AUTHOR CONTRIBUTIONS

Christopher L. Chapman, Adam W. Potter, and David P. Looney conceived and designed the research. Christopher L. Chapman, Adam W. Potter, Erica A. Schafer, J. Luke Pryor, and David P. Looney performed the experiments. Christopher L. Chapman and Adam W. Potter analyzed the data. Christopher L. Chapman, Adam W. Potter, Erica A. Schafer, Karl E. Friedl, J. Luke Pryor, and David P. Looney interpreted the results of the experiments. Christopher L. Chapman and Adam W. Potter prepared the figures. Christopher L. Chapman and Adam W. Potter drafted the manuscript. All authors edited and revised the manuscript and approved the final version.

## FUNDING INFORMATION

The research was supported by funding (MO230230) from the US Army Medical Research and Development Command (USAMRDC) Military Operational Medicine Research Program (MOMRP).

## CONFLICT OF INTEREST STATEMENT

The authors have no conflicts of interest to declare.

## DISCLAIMER

The opinions or assertions contained herein are the private views of the authors and are not to be construed as official or reflecting the views of the Army of the Department of Defense. Any citations of commercial organizations and trade names do not constitute an official Department of the Army endorsement of approval of the products or services of these organizations.

## Data Availability

The data that support the findings of this study are available from the corresponding author upon reasonable request.
